# Environmental and anthropogenic influences on movement and foraging in a critically endangered lemur species, *Propithecus tattersalli*: implications for habitat conservation planning

**DOI:** 10.1186/s40462-022-00320-x

**Published:** 2022-04-15

**Authors:** Meredith A. Semel, Heather N. Abernathy, Brandon P. Semel, Michael J. Cherry, Tsioriniaina J. C. Ratovoson, Ignacio T. Moore

**Affiliations:** 1 Department of Biological Sciences, Virginia Tech, Blacksburg, VA 24061 USA; 2 Department of Fish & Wildlife Conservation, Virginia Tech, Blacksburg, VA 24061 USA; 3 Caesar Kleberg Wildlife Research Institute, Texas A&M University-Kingsville, Kingsville, TX 78363 USA; 4 Département Zoologie et Biodiversité Animale, Université d’Antananarivo, 566 Analamanga, 101 Antananarivo, BP Madagascar

**Keywords:** Movement, Space use, Foraging, Resource selection, Brownian bridge modeling, Home range, Road avoidance, Primates, Lemurs

## Abstract

**Background:**

Wildlife conservation often focuses on establishing protected areas. However, these conservation zones are frequently established without adequate knowledge of the movement patterns of the species they are designed to protect. Understanding movement and foraging patterns of species in dynamic and diverse habitats can allow managers to develop more effective conservation plans. Threatened lemurs in Madagascar are an example where management plans and protected areas are typically created to encompass large, extant forests rather than consider the overall resource needs of the target species.

**Methods:**

To gain an understanding of golden-crowned sifaka (*Propithecus tattersalli*) movement patterns, including space use and habitat selection across their range of inhabited forest types, we combined behavior data with Dynamic Brownian Bridge Movement Models and Resource Selection Functions. We also examined the influence of abiotic, biotic, and anthropogenic factors on home range size, movement rates, and foraging patterns.

**Results:**

We found that home range size and movement rates differed between seasons, with increased core area size and movement in the rainy season. Forest type also played a role in foraging behavior with sifaka groups in the humid forest avoiding roads in both seasons, groups in the dry deciduous forest avoiding road networks in the rainy season, and groups in the moderate evergreen forest displaying no selection or avoidance of road networks while foraging.

**Conclusion:**

Our study illustrates the importance of studying primate groups across seasons and forest types, as developing conservation plans from a single snapshot can give an inaccurate assessment of their natural behavior and resources needs of the species. More specifically, by understanding how forest type influences golden-crowned sifaka movement and foraging behavior, conservation management plans can be made to the individual forest types inhabited (dry deciduous, moderate evergreen, humid, littoral, etc.), rather than the region as a whole.

**Supplementary Information:**

The online version contains supplementary material available at 10.1186/s40462-022-00320-x.

## Background

Conservation biologists have long recognized the importance of establishing protected areas to facilitate population persistence of wildlife in landscapes that are threatened by increasing human encroachment, habitat fragmentation, and habitat loss [1–4]. However, efforts to conserve wildlife and preserve biodiversity often are based on an incomplete understanding of animal movement as well as variability in movement patterns among groups or populations that the areas are meant to protect [5]. While a number of studies have demonstrated the relevance of incorporating movement, particularly animal foraging and home range size, into protected area design [6–9], integration between the disciplines of conservation biology and movement (coined “conservation behavior”) is limited [10, 11]. Yet, knowledge of movement behavior, specifically how, when, and where animals move and forage within their habitat, would illuminate how populations navigate and utilize resources within their environment and thus develop better management plans [12, 13]. Specifically, species, populations, or even groups often respond differently to factors such as seasonality, habitat characteristics, and anthropogenic pressures and therefore a better understanding of their role is crucial when developing management plans and establishing protected areas.

In many tropical regions, seasons are often divided into dry and rainy, with primary productivity varying seasonally as a function of rainfall. This seasonality thus influences the distribution and availability of resources on the landscape and as a result animal movement strategies shift to increase foraging efficiency [14–16]. For example, the black-fronted titi monkey (*Callicebus nigrifons*) [17] and collared brown lemur *(Eulemur collaris*) [18] cope with dry season food shortages by reducing movement rates, while the common bumble bee (*Bombus vosnesenskii*) [19] and African elephant (*Loxodonta africana*) [20], respond by increasing foraging and movement rates. Thus, animals are coping with dry season conditions by shifting home range size or location [21] and altering time spent foraging [22]. Understanding how seasonal fluctuations influence movement and foraging patterns in free-living animals can allow managers to more effectively design protected areas and protect critical resources [23].

In addition to abiotic factors, biotic factors such as habitat type, strongly influence animal movement and foraging [24]. Various studies demonstrate that animals adjust their home range size and foraging patterns in response to habitat type and structure (e.g. roe deer (*Capreolus capreolus*) [25] and coyote (*Canis latrans*) [26]); indicating that landscape heterogeneity is a key factor influencing the movement of species. While studies of canids, ungulates, and primates have examined the influence of habitat type on home range size, a large proportion of studies are limited to examining metrics of habitat structure (e.g., forest maturity, vegetation density, food scarcity, microhabitat preference) on animal movement and home range size [27, 28]. The benefit of understanding movement behavior across distinct habitat types is that management strategies can be designed for each habitat type a species occupies.

Importantly, anthropogenic influences affecting animal movement behaviors can have deleterious effects on wildlife, and must be considered when establishing protected areas [29, 30]. The presence of human developments and road networks may negatively influence animal movement behavior by increasing human-wildlife interactions (e.g., hunting, poaching, vehicle collisions) and pushing animals out of prime habitat [31, 32]. Large mammals may be especially affected by human encroachment due to their larger home range size, lower population density, more narrow geographic distributions, and large portions of their distributions being shared with humans [33]. For instance, black bears (*Ursus americanus*) have been found to avoid areas with human development during daylight hours [34] and woodland caribou (*Rangifer tarandus caribou*) avoid high use roads, mines, and cabins during months of high human activity [35]. Few studies have examined the influence of human infrastructure on primate movement, although they often are strongly affected by anthropogenic activities [36, 37].

The lemurs of Madagascar face significant anthropogenic threats [38]. Between 1953 and 2014, Madagascar lost 44% of its forests due to clearing for slash and burn agriculture, resulting in 46% of the remaining forests being located within 100 m of a forest edge [39]. This high degree of forest destruction and increasing presence of edge forest habitat has influenced lemur behavior and their ability to meet nutritional demands. While our understanding of lemur movement is limited, a few studies have examined lemur home range size [40, 41], dietary flexibility [42], species abundance [43], and reproduction in various forest types [44, 45]. Within the genus *Propithecus*, groups of diademed sifakas (*P. diadema*) in humid fragmented habitats had reduced home range size and daily path length and foraged on sub-optimal food items compared to sifaka groups in contiguous forest environments [40]. In contrast, groups of Milne-Edwards’ sifakas (*P. edwardsi*) inhabiting humid logged forests traveled shorter distances each day to feed in a low-quality food environment, yet maintained larger home ranges than groups in contiguous forests [41]. Further, groups of Verreaux’s sifakas (*P. verreauxi*) in Madagascar’s dry deciduous forests exhibited significant home range reduction from the rainy to the dry season [46]. While these studies have shed light on *Propithecus* behavioral responses to abiotic and biotic factors in extremes of the humid-dry forest gradient of forest types, we do not understand how species in the genus *Propithecus* respond in a moderate forest type. Knowledge of *Propithecus* movement behavior in regards to these factors would enable us to predict how these lemurs would adapt to changes in forest habitat and design a reserve accordingly.

Golden-crowned sifaka (*Propithecus tattersalli*) are a critically endangered lemur endemic to naturally fragmented forests of northeastern Madagascar [47]. Unlike the other eight species of sifaka (*Propithecus spp.*) on Madagascar that are restricted to dry or humid forest types, *P. tattersalli* inhabit a range of forest types [48, 49]. Variation of forest types they inhabit makes them a unique opportunity to examine the influence of seasonality, forest type, and anthropogenic factors on movement and foraging behavior in a primate. Studies of golden-crowned sifaka have documented a major decline in the overall population in the last decade and studies have informed researchers of the species selective use of forests in a naturally fragmented landscape. However, no studies have examined the influence of movement on space use and foraging tree selection across their range [50, 51]. An understanding of how abiotic, biotic, and anthropogenic factors influence golden-crowned sifaka space use and foraging throughout their range would allow unique management plans to be developed for distinct populations within each particular forest type occupied rather than the species as a whole.

In this study, we analyzed location and foraging behavior of six golden-crowned sifaka groups to evaluate the effects of abiotic (seasonality: rainy and dry season), biotic (forest type: dry deciduous, moderate evergreen, and humid forests), and anthropogenic (disturbance: edge and interior forests) factors on their movement patterns and space use. Approaches to studying nonhuman primate space use typically are limited to examining daily path length and home range overlap through the use of area estimators (minimum convex polygon, line-based kernel density, etc.) [52, 53]. More modern and sophisticated approaches such as Dynamic Brownian Bridge movement models (DBBMM) and Bayesian methods [54, 55] reduce the likelihood of both Type I and Type II errors which can bias our understanding of animal space use and habitat selection [56]. By selecting DBBMM to estimate space use we were able to incorporate both temporal and behavioral characteristics of movement trajectories into estimation of an animal’s home range [57]. To test our first objective, we predicted that seasonal movement rates would be greater in the rainy season, when we expected that sifakas would search out energy rich but spatially limited resources (i.e. fruits). In contrast, we expected that sifakas in the dry season would be conserving limited energy resources and thus restrict their movements. We also expected greater movement rates in more extreme forest types (dry and humid forests) and edge forests compared to moderate evergreen and interior forests for similar energetic reasons [58], as well as differences in sifakas densities between the three forest types [59]. Second, we predicted that home range size and core area range size would be larger in the rainy season compared to the dry season, as sifakas would maximize their foraging area to exploit energy rich, rainy season resources. We also expected home range and core areas to be larger along forest edges, where energy-rich resources may be more limited [58]. Additionally, we predicted that home range and core areas sizes would be larger in the dry and humid forests where golden-crowned sifaka densities are lower, and smaller in the moderate forests where golden-crowned sifaka densities are higher [59]. Third, we predicted that sifakas would select foraging locations with the largest feeding trees within their home ranges, which we expected to have the most available fruits [60], and avoid locations near human settlements or man-made structures, where we expected forest disturbance to be greatest [61].

## Methods

### Study area

**Fig. 1:**
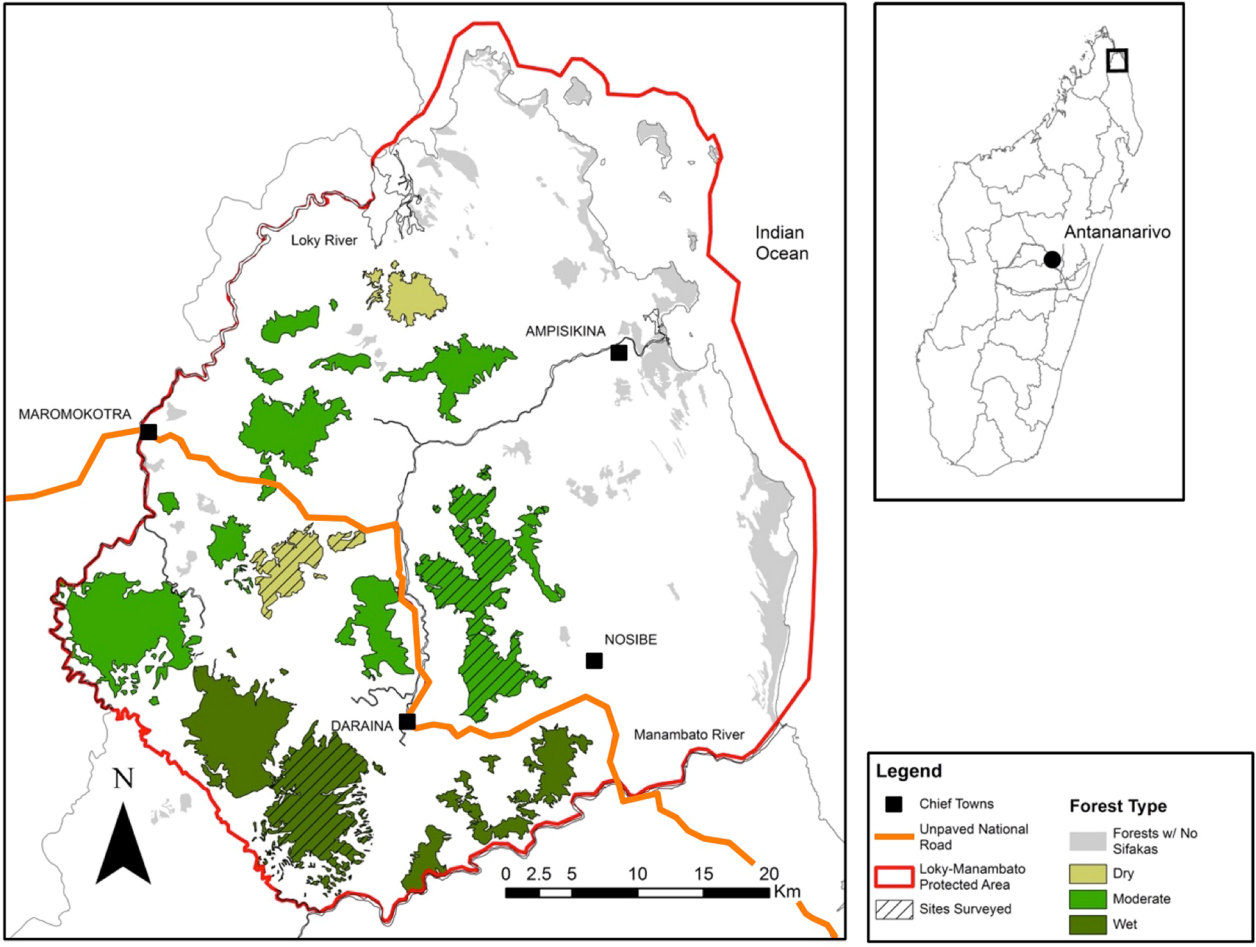
Map of the golden-crowned sifaka (*Propithecus tattersalli*) range within the Loky-Manambato Protected Area in northeastern Madagascar, as indicated in the box on the inset of Madagascar. Different shades of green indicate the three main forest types and hatched black lines indicate the three forest fragments surveyed: dry (light green, Solaniampilana), moderate (green, Bekaroaka), and wet (dark green, Binara). The thin orange line depicts the unpaved national road in the region

Research was conducted in the Loky-Manambato Protected Area (49°56ʹE, 13°31ʹS) of northeastern Madagascar (Fig. 1). This protected area encompasses a unique biogeographical transition zone from Madagascar’s northern and western dry deciduous forests to southern humid forests. The Loky-Manambato region contains a mosaic of various forest types including dry deciduous, dry evergreen, humid, and littoral forests separated by agricultural areas and savanna [50]. The total forest cover of this protected area is 475.3 km^2^ and individual forest fragments range from 11.6 to 46.3 km^2^. The region experiences a 4-month rainy season from December to March followed by an 8-month dry season [62]. The study sites include three distinct forest types: humid forest, moderate evergreen forest, and dry deciduous forest.

### Study species and subjects

Golden-crowned sifaka live in semi-cohesive social groups ranging in size from 3 to 12 individuals with one or more adult males, several adult females, and several immature individuals of both sexes. Group members typically travel in a coordinated fashion and generally remain in visual or auditory contact with at least one other group member [62]. Thus, we assume that all animals within a given social group share a home range, and therefore treated each group as a distinct unit of analysis. Golden-crowned sifaka are frugo-folivores, but also consume seeds, petioles, buds, flowers, and bark.

We studied six groups of golden-crowned sifaka distributed across the three distinct forest types (two groups each in dry deciduous, moderate evergreen, and humid forest) in the Loky-Manambato Protected Area. Prior to data collection, all groups were habituated to human presence. Habituation was considered complete when lemurs no longer alarm called, fled from human presence, or moved closer to observers out of curiosity. Similar to studies conducted with other lemur species, this process took less than 2 months [63]. We selected three of the 11 large forest fragments containing golden-crowned sifaka due to their accessibility: Solaniampilana (dry deciduous), Bekaraoka (moderate evergreen), and Binara (humid) (Fig. 1). Sifaka densities within these fragments were variable with 26.7 sifakas/km^2^ (95% CI 16.2–44.1) in Solaniampilana, 78.17 sifakas/km^2^ (95% CI 53.1–114.8) in Bekaraoka, and 20.77 sifakas/km^2^ (95% CI 11.2–38.0) in Binara [59]. Within each forest type, we followed one group in primary forest towards the center of the forest (hereafter interior; characterized by lemurs having a home range at least 300 m from the forest edge) and one group on the edge of the forest fragment (hereafter edge; characterized by having a home range adjacent to the forest edge). Average group size was six individuals and ranged from five to eight (Table 1). Although lemur groups were not marked or collared, we identified individual lemurs by their distinct, permanent physical features (e.g. missing eye, ear cuts, coloration) to ensure we were following the target group.

**Table 1:** Composition of golden-crowned sifaka (*Propithecus tattersalli*) focal groups within each forest fragment, fragment size, forest type, and forest disturbance classification of the Loky-Manambato protected area in northeastern Madagascar

Forest fragment	Fragment size (km^2^)	Forest type	Forest location	Group size
Solaniampilana	14.7	Dry deciduous	Interior	5
Solaniampilana		Dry deciduous	Edge	5
Bekaraoka	26.2	Moderate evergreen	Interior	7
Bekaraoka		Moderate evergreen	Edge	8
Binara	43.6	Wet humid	Interior	7
Binara		Wet humid	Edge	5

Groups were followed during the dry (June–August 2019) and rainy (February–April 2019) seasons

### Group location data

We collected golden-crowned sifaka group location data during two periods, February-April 2019 (rainy season) and June–August 2019 (dry season). Each group was followed for 7–9 consecutive days during the rainy season and the dry season. We followed groups from sleep tree to sleep tree (~ 12 h per day) and collected location data at 15-min intervals. If no animals were visible at the 15-min interval, observers waited to establish visual contact with the social group before recording any locations. During behavioral follows, we maintained a distance of at least 10 m from the lemurs and followed slowly behind the groups in an effort to minimize disrupting their natural behaviors. While the use of telemetry based tracking technology has been shown to reduce disturbances to wildlife behavior, we chose behavioral follows due to a lack of tracking equipment but also to avoid subjecting the animals to the stress of capture and immobilization [64]. In addition to daytime activity, golden-crowned sifaka are known to exhibit nocturnal movements, specifically during periods of bright moon light [65], and thus groups were not always located in the same sleep tree the following morning. In those instances, we reestablished contact with the group as quickly as possible. Group locations were recorded using a GPS receiver (Garmin 64 s), using the Universal Transverse Mercator (UTM) coordinate system (zone 39L), and points were logged at the group’s approximate geometric center.

### Foraging and landscape data

We recorded foraging data at the same 15-min intervals using scan sampling to record the behavior, height in the tree, and nearest neighbor of each individual in a group [66]. If an individual was actively feeding during the scan, the plant species and part (e.g., young/mature leaf, leaf petiole, un/ripe fruit, seed, or flower) were identified, GPS location recorded, and data concerning tree species, size, and current phenology collected. In addition to collecting foraging data specific to each of the lemur groups, we also collected general landscape data throughout each of the six lemur home ranges in both the rainy and dry season. We did this by randomly generating forty GPS points within each of the six home ranges (in both rainy and dry seasons) and collected data from potential feeding trees (species, size, phenology) within five m of each location. This allowed us to gain an understanding of the entire landscape of all six home ranges, not just the specific feeding trees utilized by each of the groups.

### Home range estimation

Utilization distributions (i.e., 95% isopleth, hereafter home ranges and 50% isopleth, hereafter core area) were estimated for each golden-crowned sifaka group using Dynamic Brownian Bridge Movement Models (DBBMM); [57]. Home range DBBMMs use behavior and movement trajectory data of the animal group that is collected in sequential relocation studies. This method provides a spatially explicit model, which describes the probability of the given animal group occurring in a given location during a specified period. This approach also accounts for temporal autocorrelation, spatial uncertainty, irregularly sampled data, and shifts in an animal’s behavior (resting, foraging, thermoregulating, corridor use, etc.), making it specifically applicable to studies of group living primates [56, 57, 67]. Using DBBMMs to estimate group home ranges requires a Brownian motion variance parameter (σ^2^, in meters), which quantifies the degree of diffusion or irregularity of an animal’s path [57]. A moving window analysis identifies changes in the movement behavior and estimates σ^2^ for each step. Because the σ^2^ parameter is estimated using a “leave-one-method”, the size of the moving window must include an odd number of GPS locations and a margin of greater than three locations bounding each end of the window in which no behavioral changes can occur [57]. We parameterized the DBBMM with a 21-step window size, a 9-step margin size, and a 15 m location error for all lemur groups, as visual inspection indicated these settings were sufficient to identify changes in home range size and overall animal movement [57]. Home ranges were estimated for each lemur group using the DBBMM function in R package ‘move’ [68, 69]. We conducted a three-way analysis of variance (ANOVA) predicting for both home range and core areas sizes, respectively, to determine if season, forest type, interior or edge forests, and the interaction of forest type and season influenced core area and home range size. All analyses were conducted in version 3.6.1 of program R [70]. We used Akaike’s Information Criterion corrected for small samples sizes (AICc) to identify a top model from the set of candidate ANOVA models and the delta of two was as a threshold for equally plausible models [71].

### Seasonal core area overlap

To determine the percent of joint home range overlap between the rainy and dry season core areas, we calculated the total area of each home range and then divided the area of overlap between seasons by the total home range size [72].

$$\left[ {\left( {{\text{area}}_{{\upalpha \upbeta }} /{\text{core - area}}_{\upalpha } } \right) \times \left( {{\text{area}}_{{\upalpha \upbeta }} /{\text{core - area}}_{\upbeta } } \right)} \right]^{0.5}$$

Area_αβ_ is the seasonal core area overlap area common to α and β, and core-area α and core-area β are the seasonal core areas of the same group during the rainy and dry season, α and β, respectively. Possible core area overlap ranged from 0% overlap, indicating no shared space use between seasons, to 100% overlap, indicating the dry and rainy season core area ranges overlapped completely. To determine if the core area overlap between the rainy and dry seasons varied as a function of forest type (humid, moderate, or dry), we conducted a one-way ANOVA comparing core area overlap as a function of degree of forest type. All analyses were conducted in version 3.6.1 of program R [70].

### Movement rates

We calculated movement rates (meters/monitoring interval [15-min]) for each lemur group using the collected relocation data. The step length (i.e., the distance between sequential recorded locations) was divided by the time elapsed between each sequential location (15-min) to calculate speed for each golden-crowned sifaka group to characterize movement rates. To determine how movement varied across season (rainy and dry), forest disturbance (edge or interior), and forest types (dry, moderate, and humid) we calculated movement rates at both the daily and seasonal scale.

Daily movement rates were bootstrapped to calculate a mean for each observational day. Bootstrapping is a process that involves repeatedly drawing independent samples from a data set (x) to create bootstrap data sets (x^1^, x^2^,…, x^n^). Our samples were performed with replacement which allowed for the same observation to be sampled more than once such that each bootstrapped sample was the same length as our raw lemur speed data (m/15-min). To calculate seasonal movement rates ($\widehat{SMR}$), we drew 1000 independent samples (${\widehat{\alpha }}^{1},{\widehat{\alpha }}^{2},\dots ,{\widehat{\alpha }}^{B})$ to calculate means and standard error (${\widehat{SE}}_{B}$), which we then used to generate 95% confidence intervals for comparison of means among seasons and groups,

$$\widehat{SE}_{B} = \sqrt {\frac{{\mathop \sum \nolimits_{b = 1}^{B} \left( {\hat{\alpha }^{i} - \hat{\alpha }^{B} } \right)^{2} }}{{\left( {B - 1} \right)}}}$$

where ${\widehat{SE}}_{B}$ served as our estimate of the standard error of $\widehat{\alpha }$ estimated from the raw lemur speed data (m/h). We calculated seasonal movements rates using the bootstrapping approach outlined above but employed the method for each observation season [73, 74].

To determine how environmental variables influenced daily movement rates, we fit LMMs to predict movement rate as a function of all combinations of season (rainy or dry), forest disturbance (edge or interior), and forest type (dry, moderate, and humid), while treating forest type-forest fragmentation per group intercepts as random effects [75]. We used Akaike's Information Criterion corrected for small sample size (AICc) to identify a top model from the set of candidate models [76]. We used the Satterthwaite method to approximate the degrees of freedom and computed *p*-values for direct effects and interactions using t-statistics [77].

Finally, to determine how environmental variables influenced seasonal movement rates, we conducted a three-way ANOVA of seasonal movement rates as a function of forest type (dry, moderate, and humid), forest disturbance (edge and interior), and season (dry and rainy). We used Akaike's Information Criterion corrected for small sample size (AICc) to identify a top model from the set of candidate models [76]. All analyses were conducted in version 3.6.1 of program R [70].

### Habitat selection

To quantify habitat selection of golden-crowned sifaka groups, in relation to tree size and proximity to anthropogenic factors, we fit a Resource Selection Function (RSF) using a use-available design. A RSF is defined as any function producing a value proportional to the probability of selection of a given habitat [78, 79]. Any estimate derived from an RSF is dependent on the definition of available habitats [54, 78, 80]. For our RSF, selection by golden-crowned sifaka availability was considered within home range selection (Johnson’s third order [80]) as defined by a 95% seasonal home range using DBBMMs. Within our seasonal home ranges, we characterized availability by systematically identifying available locations at intervals of 10 m, as this was the spatial resolution of all spatial data used in the RSF [81].

We created our RSFs by fitting generalized linear mixed-effects model (GLMM) with a binomial exponential family and logit link function, which included a group-specific (forest type and disturbance) random intercept term to account for non-independence of habitat associations within groups [82, 83]. For our RSF, we used GPS locations of all feeding trees that golden-crowned sifaka utilized during the rainy and dry field seasons and possible locations within their known home ranges. We extracted tree basal area (cross-sectional area of trees at breast height), Euclidian distance to village, road, and habitat fragment edge for each golden-crowned sifaka feeding tree and each available location. These data were generated using satellite imagery and habitat sampling of resources within each of the six lemur home ranges.

To relate tree basal area and crown volume to lemur GPS location data, we created continuous surfaces of tree basal area and crown volume estimates across our study area by using inverse distance weighting (IDW) interpolation in the package gstat [84]. IDW uses a weighted average of estimates from nearby sampling locations to predict tree basal area and crown volume estimates to the surrounding pixels of a sampling location composed of user-specified areas [85]. Our user-specified areas of inference were 169 m^2^ because it most closely matched the mean distance between vegetation sampling locations (148.85 m). This interpolation process provided spatially explicit estimates of tree basal area and crown volume estimates which we could then associate with our lemur GPS data.

To examine if lemur habitat selection varied across forest types and seasons, we developed candidate models using various combinations of distance to habitat feature (i.e., village, road, and habitat fragment), and basal area, and used Akaike's Information Criterion (AICc) corrected for small samples sizes to identify a top model from the set of candidate models [71] to determine if (1) differences in habitat selection vary as a function of forest type, and (2) differences in habitat selection vary as a function of season at each site. To account for behavioral differences in lemur groups, we accounted for random effects using an ‘animal ID’ that consisted of each lemur group’s respective forest type (dry, moderate, or humid), forest disturbance classification (edge or interior), and season (rainy or dry). We tested for collinearity and no environmental variables used in model development exhibited high correlation (i.e., |r|> 0.7). All coefficients were estimated using the “lme4” package [70, 75].

Overdispersion of the models was examined by calculating the sum of squared Pearson (SSQ) residuals, the ratio of (SSQ residuals/residual degrees-of-freedom), the residual *df*, and the *p*-value based on the appropriate χ^2^ distribution. Additionally, each individual model was a GLM which allowed us to test overdispersion using a chi-squared comparing the model deviance by the residual degrees-of-freedom [86]. To estimate the explanatory power of the models, we calculated a conditional, marginal, and pseudo R^2^ for each model [87, 88].

## Results

### Home range and core area size estimations

Overall, home range sizes for golden-crowned sifaka groups in the Loky-Manambato Protected Area were highly variable, ranging from 2.78 to 31.56 hectares (Table 2). Our top ANOVA model (Table 3), revealed that golden-crowned sifaka core areas varied significantly with season (*p* = 0.006, F(1,10) = 11.84, residual SE = 0.005) with core areas being larger in the rainy season (average of 1.74 hectares in the rainy season, 0.81 hectares in the dry season). However, while our ANOVA model candidate set for home ranges did include season as a top model (dry or rainy; *p* = 0.136, F(1,10) = 2.63, SE = 0.07), it was no better than our null model (Table 3).

**Table 2:** Data summary of six golden-crowned sifaka (*Propithecus tattersalli*) groups followed in different forest types (dry, moderate, and humid) in three forest fragments (Solaniampilana, Bekaraoka, Binara) of two levels of forest disturbance (interior or edge) in the Loky-Manambato Protected Area in northeastern Madagascar

Lemur group ID	Number of follow days		Number of GPS locations		Home range (ha)		Core area (ha)		Core Area (% overlap)
	Dry	Rainy	Dry	Rainy	Dry	Rainy	Dry	Rainy	
Dry-interior	9	7	285	206	6.04	7.95	0.88	1.91	16.7%
Dry-edge	8	7	270	216	8.19	31.6	0.89	2.62	51.2%
Moderate-interior	8	9	266	334	2.93	9.16	0.63	1.87	41.0%
Moderate-edge	8	8	299	269	2.78	4.83	0.57	0.78	16.3%
Humid-interior	8	9	282	320	3.69	25.1	0.68	1.71	32.0%
Humid-edge	9	8	290	278	12.5	11.1	1.18	1.90	53.9%

The number of consecutive follow days, the number of GPS locations recorded, home range size (hectares), and core area size (hectares) are indicated for both the dry (June–August 2019) and rainy (February–April 2019) seasons. Percent core area overlap between seasons also is included

**Table 3:** Linear mixed effects models (LMMs) to explain core area size and home range size of golden-crowned sifaka (*Propithecus tattersalli*) groups in the Loky-Manambato Protected Area in northeastern Madagascar during the dry (June–August 2019) and rainy (February-April 2019) seasons

Model	K	LL	AICc	ΔAICc	W
*Core area size*					
Season	1	48.407	− 87.8	0.00	0.816
Disturbance + season	2	48.484	− 83.3	4.56	0.083
Null	0	43.720	− 82.1	5.71	0.047
Forest type + season	3	50.968	− 81.9	5.88	0.043
Disturbance	1	43.755	− 78.5	9.30	0.008
Forest type	2	44.759	− 75.8	12.01	0.002
Forest type + season + disturbance	4	51.086	− 73.4	14.44	0.001
Forest type + disturbance	3	44.801	− 69.6	18.21	0.000
*Home range size*					
Null	0	13.999	− 22.7	0.00	0.465
Season	1	15.398	− 21.8	0.87	0.301
Disturbance	1	14.558	− 20.1	2.55	0.130
Disturbance + season	2	16.113	− 18.5	4.15	0.058
Forest type	2	15.538	− 17.4	5.30	0.033
Forest type + season	3	17.417	− 14.8	7.83	0.009
Forest type + disturbance	3	16.271	− 12.5	10.12	0.003
Forest type + disturbance + season	4	18.443	− 8.1	14.58	0.000

Season, disturbance, and forest type were included in the model. Columns indicate the number of parameters (K), log-likelihood (LL), the relative difference in AICc values compared to the top-ranked model (ΔAICc), and the AICc model weights (W) of the model-selection procedure

### Seasonal core area overlap

**Fig. 2:**
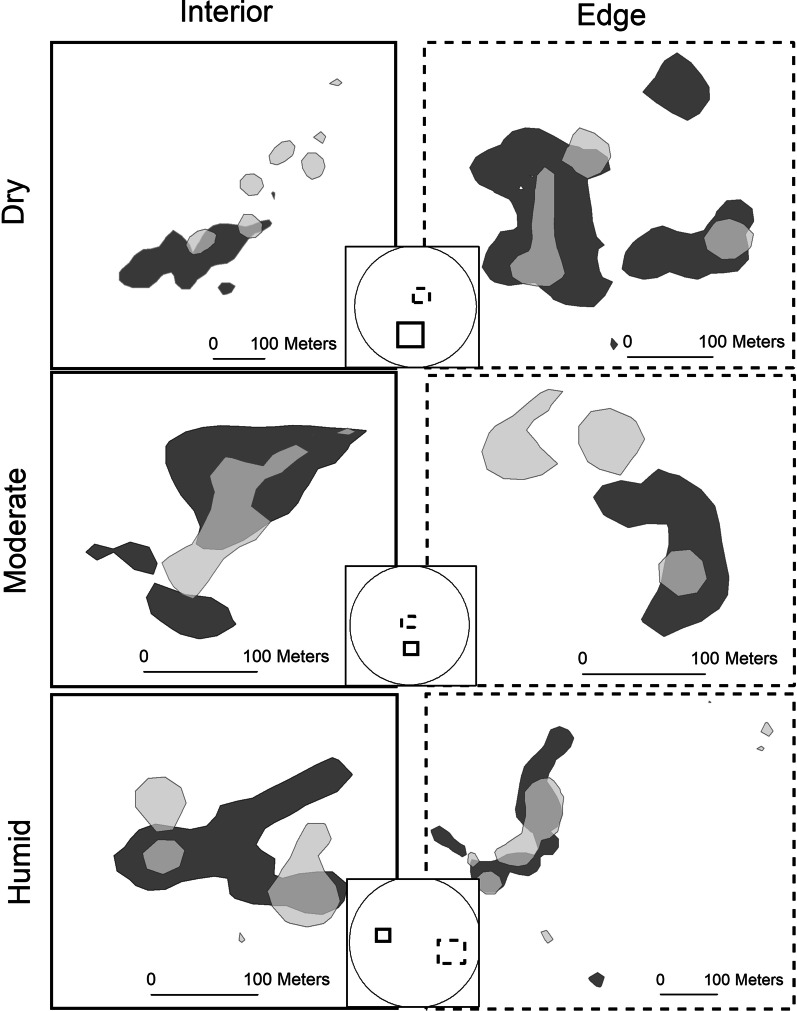
Maps of the Brownian Bridge utilization distributions depicting core area use for golden-crowned sifaka (*Propithecus tattersalli*) groups in the Loky-Manambato Protected Area of northeastern Madagascar during the dry (June–August 2019; dark gray) and rainy (February-April 2019; light gray) seasons. Overlapping areas were occupied during both seasons. The six boxes display the seasonal home ranges for all six lemur groups followed. Columns indicate forest disturbance classification (interior or edge) and rows indicate the occupied forest type (dry, moderate, or humid). Solid lines correspond to the interior and the hashed lines correspond to the edge in the inset map of each site

Seasonal core area overlap varied from 16.7 to 53.9% (Table 2; Fig. 2). Core area overlap between the rainy and dry season did not vary with forest type (*p* = 0.824, F(2.3) = 0.214, SE = 0.072) or forest disturbance (*p* = 0.617, F(1.4) = 0.343, residual SE < 0.091).

### Daily and seasonal movement rates

**Fig. 3:**
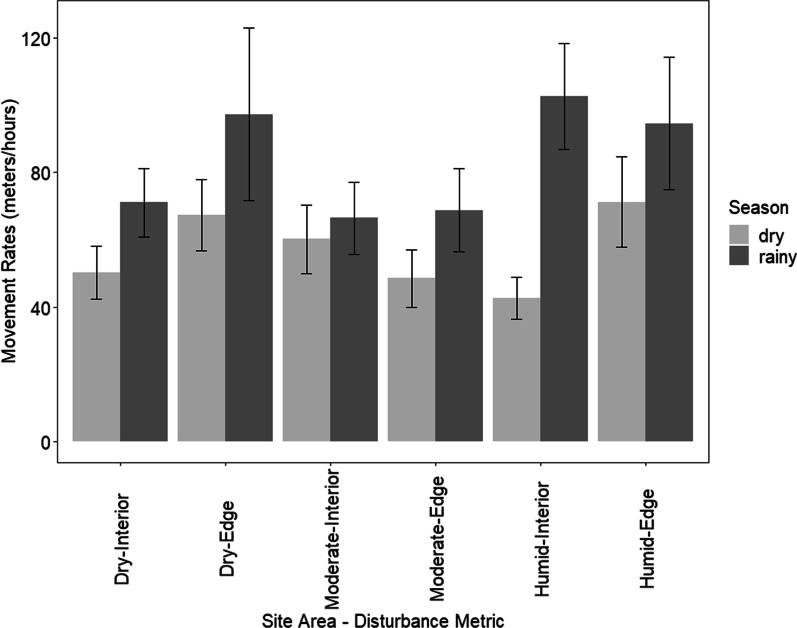
Seasonal movement rates (meters/hour) of golden-crowned sifaka (*Propithecus tattersalli*) groups in the Loky-Manambato Protected Area in northeastern Madagascar. Data was collected during the dry (June–August 2019) and rainy (February-April 2019) seasons using relocation data collected every 15 min. The step length (e.g., the distance between sequential locations) was divided by the time elapsed between each step to calculate speed for each lemur group. Black lines correspond to 95% confidence intervals

Our top model (Table 4) indicated that seasonal movement rates varied as a function of season (Sum Squares = 0.405, 95% CI [0.13, 0.65], F(1,10) = 11.27, *p* = 0.007, residual S.E. = 0.04), with higher rates in the rainy season (rainy season: 83.47 m/h; dry season: 56.70 m/h; Fig. 3; Additional file 1: Table S1). When investigating daily movement rates, our LMM analysis supported our seasonal movement results, as the top model included season; however, there was not support for effects of forest type or forest disturbance (β = 0.36, 95% CI [0.11, 0.60]; *p* = 0.019; Table 4; Additional file 1: Table S2).

**Table 4:** Competing models to explain seasonal and daily movement rates of golden-crowned sifaka (*Propithecus tattersalli*) groups in the Loky-Manambato Protected Area in northeastern Madagascar during the dry (June–August 2019) and rainy (February–April 2019) seasons

Model	K	LL	AICc	ΔAICc	W
*Seasonal movement rates*					
Season	1	3.382	2.2	0.00	0.733
Disturbance + season	2	4.394	4.9	2.69	0.191
Null	0	− 1.145	7.6	5.39	0.050
Disturbance	1	− 0.691	10.4	8.15	0.012
Forest type + season	2	4.779	10.4	8.21	0.012
Forest type	2	− 0.529	14.8	12.54	0.001
Forest type + season + disturbance	4	6.086	16.6	14.39	0.001
Forest type + disturbance	3	− 0.023	20.0	17.81	0.000
Forest type + season + forest type*season	5	6.158	29.7	27.45	0.000
Forest type + disturbance + season + forest type*season	6	7.856	48.3	46.05	0.000
*Daily movement rates*					
Season	1	− 27.381	63.3	0.00	0.586
Null	0	− 29.393	65.1	1.82	0.236
Disturbance + season	2	− 28.186	67.1	3.87	0.085
Disturbance	1	− 30.082	68.7	5.40	0.039
Forest type + season	2	− 28.022	69.1	5.87	0.031
Forest type	1	− 30.101	71.0	7.70	0.012
Forest type + season + disturbance	3	− 28.845	73.2	9.89	0.004
Forest type + season + forest type*season	4	− 27.815	73.6	10.28	0.003
Forest type + disturbance	2	− 30.777	74.6	11.38	0.002
Forest type*season + disturbance	5	− 28.634	77.7	14.43	0.000

Season, disturbance, forest type, and all interactions (*) were included in the model. Columns indicate the number of parameters in the model (K), the log-likelihood (LL), the relative difference in AICc values compared to the top-ranked model (ΔAICc), and the AIC model weights (W) of the model-selection procedure

### Habitat selection

We found that lemur habitat selection varied between humid, moderate, and dry forest types as indicated by AICc (Table 5). We also found that selection by lemurs varied between the wet and dry season, within forest types, as indicated by AICc (Table 6). Given selection varied by forest type and season, we elected to make inferences on selection by forest type and by season. This necessitated we change our models from GLMMs to generalized linear models (GLMs) wherein we dropped the group-specific (forest type and disturbance) random intercept term given our final models retained only two groups [89]. Tests for overdispersion and explanatory power raised no concern (Additional file 1: Tables S3 and S4).

**Table 5:** Generalized linear models (GLMs) of golden-crowned sifakas (*Propithecus tattersalli*) depicting differences in foraging tree selection among lemurs occupying different forest types in the Loky-Manambato Protected Area in northeastern Madagascar during the dry (June–August 2019) and the rainy (February–April 2019) seasons

	K	AICc	ΔAICc	W	LL
(CV + TBA + V + R + F)*FT	19	11,353.62	0.00	1.00	− 5657.81
CV + TBA + V + R + F	7	11,425.18	71.56	0.00	− 5705.59

Columns indicate the number of parameters (K), the relative difference in AICc values compared to the top ranked model (ΔAICc), the AICc weights (W), and the log-likelihood (LL) of the model-selection procedure examining foraging tree selection of lemurs based on occupied forest type (humid, moderate, and dry). CV: Crown Volume, TBA: Tree basal area, V: Distance to village, R: Distance to roads, F: Distance to forest edge, FT: Forest type. Based on the models, forest types could not be grouped and were parsed to make assumptions

**Table 6:** Generalized linear models (GLMs) of golden-crowned sifakas (*Propithecus tattersalli*) foraging tree selection based on occupied forest type in the Loky-Manambato Protected Area in northeastern Madagascar during the dry (June–August 2019) and rainy (February–April 2019) seasons

	K	AICc	ΔAICc	W	LL
*Dry forest*					
(CV + TBA + V + R + F)*Season	13	4085.42	0.00	1.00	− 2029.71
CV + TBA + V + R + F	7	4105.11	19.7	0.00	− 2045.56
*Moderate forest*					
(CV + TBA + V + R + F)*Season	13	2944.24	0.00	1.00	− 1459.12
CV + TBA + V + R + F	7	3043.27	99.04	0.00	− 1514.64
*Humid forest*					
(CV + TBA + V + R + F)*Season	13	4158.31	0.00	1.00	− 2066.15
CV + TBA + V + R + F	7	4206.98	48.68	0.00	− 2096.49

Columns indicate the number of parameters (K), the relative difference in AICc values compared to the top ranked model (ΔAICc), the AICc weights (W), and the log-likelihood (LL) of the model-selection procedure examining foraging tree selection of lemurs based on occupied forest type (dry, moderate, and humid). CV: Crown Volume, TBA: Tree basal area, V: Distance to village, R: Distance to roads, F: Distance to forest edge, FT: Forest type

**Fig. 4:**
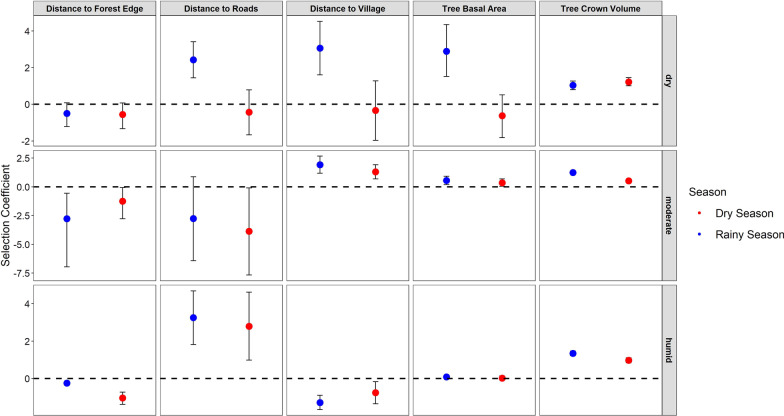
Selection coefficient plot for golden-crowned sifakas (*Propithecus tattersalli*) in the Loky-Manambato Protected Area in northeastern Madagascar in the dry (June–August 2019) and rainy (February-April 2019) seasons within three forest types (dry, moderate, and wet). This coefficient plot displays beta estimates for tree basal area and tree crown volume and distance to forest edge, roads, and villages. Blue points represent habitat selection during the rainy season and red points represent habitat selection during the dry season. Solid lines above and below each point represent the 95% confidence intervals around each beta estimate

We found that groups in dry deciduous forests selected locations with greater crown volume in the dry season (β = 1.22, S.E. ± 0.11, *p* < 0.001). During the rainy season, groups in dry forest selected locations with greater crown volume (β = 1.04, S.E. ± 0.12, *p* < 0.001) and greater tree basal area (β = 2.89, S.E. ± 0.73, *p* < 0.001), and avoided habitat closer to villages (β = 3.06, S.E. ± 0.74, *p* < 0.001) and roads (β = 2.42, S.E. ± 0.50, *p* < 0.001; Fig. 4).

Lemur groups in moderate evergreen forests selected locations with greater crown volume (β = 0.52, S.E. ± 0.07, *p* < 0.001), greater tree basal area (β = 0.35, S.E. ± 0.17, *p* = 0.03), and locations farther from villages (β = 1.30, S.E. ± 0.32, *p* < 0.001) in the dry season. In the rainy season, groups selected feeding locations with greater tree crown volume (β = 1.24, S.E. ± 0.07, *p* < 0.001) and greater tree basal area (β = 0.55, S.E. ± 0.19, *p* = 0.003) and avoided habitats closer to villages (β = 1.91, S.E. ± 0.38, *p* < 0.001; Fig. 4).

Finally, we found that lemur groups in the humid forests selected feeding locations characterized with greater crown volume (β = 0.98, S.E. ± 0.07, *p* < 0.001), closer to villages (β = -0.76, S.E. ± 0.30, *p* = 0.01), and closer to the forest edge (β =  − 1.04, S.E. ± 0.17, *p* < 0.001), and avoided locations near roads (β = 2.79, S.E. ± 0.93, *p* = 0.003) in the dry season. In the rainy season groups in humid forests selected locations with greater tree basal area (β = 0.085, S.E. ± 0.04, *p* = 0.049) and greater crown volume (β = 1.34, S.E. ± 0.06, *p* < 0.001) that were closer to villages (β = -1.28, S.E. ± 0.19, *p* < 0.001), and forest edges (β =  − 0.24, S.E. ± 0.05, *p* < 0.001), and avoided habitats near roads (β = 3.26, S.E. ± 0.73, *p* < 0.001). While the effects of crown volume, villages, forest edges and roads were the same across seasons, these effects were stronger in the rainy season (Fig. 4).

## Discussion

There are three primary results from our study. First, golden-crowned sifaka (*Propithecus tattersalli*) movement rates are greater in the rainy season and in more humid forests. Second, golden-crowned sifaka core area size is impacted by season, with larger core areas used in the rainy season. Third, golden-crowned sifakas select foraging locations where the largest trees in their home range are located. Similarly, we also detected variation in behavioral responses to villages, road networks, and the forest edge. Golden-crowned sifaka groups in humid and dry deciduous forest fragments specifically avoided foraging locations near road networks in the rainy season, while lemurs in the moderate evergreen forest did not select or avoid locations near road networks. In sum, groups of golden-crowned sifaka showed marked variation in behavioral responses to human disturbance, but for all groups, higher-use zones were characterized by locations having larger trees. Thus, season, forest type, and disturbance all have effects on golden-crowned sifaka space use and ranging behavior.

Across seasons and regardless of forest type or disturbance, golden-crowned sifaka group daily movement rates shifted, with groups moving farther per unit time in the rainy season. This contrasts with previous studies on Milne-Edwards’ sifaka groups that found no seasonal effects on distance moved per day (i.e., daily path length) [41]. Thus, there exists some degree of variability among sifaka species. Movement rates in golden-crowned sifaka groups were also closely linked to home range size in that as home range size increased in the rainy season, so did the average distance moved per hour. This finding is consistent with other studies of highly mobile mammals that found that movement rates and resource availability determined home range size of white-tailed deer (*Odocoileus virginianus*) and Iberian ibex (*Capra pyrenaica*) [90, 91].

Golden-crowned sifaka group home range sizes varied between 3 and 32 ha. These home range sizes were smaller than those of diademed sifaka groups in Madagascar’s eastern humid forests, which range from 19 to 79 ha [92], but were larger than those of Verreaux’s sifaka groups in Madagascar’s southern dry forests, which have home range sizes ranging from 5 to 10 ha [93]. Similar to this trend, mouse lemurs (*Microcebus* spp) inhabiting western dry forests were able to maintain higher population densities than mouse lemur species inhabiting eastern humid forests [94]. Contrary to previous findings, and our predictions, we found that golden-crowned sifaka groups in dry deciduous and humid forests did not occupy significantly larger home range or core area sizes compared to groups living in moderate evergreen forest fragments. This finding was unexpected because golden-crowned sifaka densities were substantially larger in moderate forest fragments compared to dry deciduous and humid forest fragments [59]. In sum, our study was the first determining that the same species of sifaka can inhabit drastically different forest types and display great variation in home range size. However, while animal densities have implications for spatial ecology [95], golden-crowned sifaka densities did not appear to predict home range and core areas sizes [59].

Our prediction that golden-crowned sifaka home range sizes would vary between the rainy season and the dry season was partially supported. While home range sizes were not significantly different between seasons, core area size was statistically larger for golden-crowned sifaka groups in the rainy season compared to the same groups’ core area range sizes in the dry season. Similar to findings of Milne-Edwards’ sifaka, we found that golden-crowned sifaka maintained similar home range locations in both seasons, but displayed considerable seasonal shifts in core area locations [41]. This difference was likely due to the non-uniform and seasonal variation in resource distribution that influenced how golden-crowned sifaka distributed their space use to forage efficiently [96]. Surprisingly, the degree of core area overlap we observed did not vary based on the forest type or forest disturbance level occupied.

We predicted that golden-crowned sifaka groups in more degraded, edge, habitats would occupy larger home ranges and have larger core areas than those in interior forests; however, our data did not support that assumption. Previous studies have demonstrated varying effects of disturbance on home range size of eastern sifaka species in rainforest habitats. For instance, diademed sifaka living along forest edges occupied significantly smaller home range sizes than conspecifics in interior, undisturbed forests [40], as is consistent with many other mammalian species [97]. However, Milne-Edwards’ sifaka in disturbed (logged) forests maintained larger home range sizes than those in undisturbed forests [41]. Unfortunately, the majority of lemur studies (87%) examining the effects of forest disturbance on lemur health, genetics, biodiversity, and behavior were conducted in the humid forests of eastern Madagascar [98]. Further, lemur responses to habitat edges in dry forest are often highly variable, with groups avoiding, selecting, or demonstrating no response in regards to feeding along forest edges [98, 99]. As a result, further investigation of home ranges of golden-crowned sifaka and other dry forest lemurs are needed to understand how increasing anthropogenic changes are influencing lemur ecology and conservation.

While we expected golden-crowned sifaka groups to avoid villages and roads, we found a mixed response. In some forest types and seasons, sifaka groups selected for foraging locations near roads, while in other forest types and seasons sifaka groups avoided foraging locations near roads. The same mixed response was found in regard to foraging locations near villages. Other studies have found similar mixed responses to anthropogenic influences [100, 101]. Additionally, the national road that bisects the global range of golden-crowned sifaka is currently being paved to improve access to mineral reserves and transportation through the region. While infrastructure improvement will likely provide much needed improvement to local economies, our results demonstrate that even narrow road networks can restrict suitable sifaka habitat. Improved road access also is likely to increase resource extraction within the region (e.g., selective logging of hardwoods and gold mining), with negative impacts expected for sifakas [38]. The negative impacts of road networks are well documented for mammalian species. For example, elk (*Cervus canadensis*) and caribou (*Rangifer tarandus*) tend to avoid road crossings and seek cover when in close proximity to road networks [102]. Road expansion and paving also increases the prevalence of vehicle collisions with wildlife (e.g., Asiatic cheetah (*Acinonyx jubatus venaticus*) [103] and Florida panther (*Puma concolor coryi*)) [104]. Importantly, even low vehicle traffic (0–30 vehicles/12 h) caused wolverines (*Gulo gulo*) to alter their movement patterns and to avoid areas with road networks [105]. Consequently, increasing human activity and road prevalence is likely to impact foraging and space use behavior of wildlife species, with potentially disastrous consequences for already crucially endangered golden-crowned sifaka.

## Conclusions

### Limitations and future directions

Although our data revealed various patterns in golden-crowned sifaka movement and foraging patterns, we could only establish general conclusions due to our limited monitoring efforts (15–18 days/year for each group) and small sample size (six sifaka groups). Additionally, due to our rotation-based field schedule, we were only able to follow one group of sifaka at a time. Future work with more continuous monitoring, preferably with the use of remote tracking devices, would enable us to better tease apart the relationships between season, forest type, anthropogenic factors, and movement, as well as to increase model robustness. Second, there were limitations regarding the covariates used in the RSF. Specifically, we assumed that all village disturbance was equal. However, each village differed in population size, degree of infrastructure, and distance from the forest edge. As a result, some villages could have had greater anthropogenic influences than others. Third, we could not claim that human disturbance was directly related to golden-crowned sifaka groups selecting for the largest trees within their respective home ranges. Feeding on the largest trees could have been related to increased food availability and security, rather than human driven factors. Fourth, we used simple categories (e.g., ‘rainy’ and ‘dry’) to reflect seasons, forest types, and disturbance levels. While this uncovered broad patterns, more sophisticated resource measurements, such as precipitation amounts or Landsat imagery, would improve the context within which we interpret sifaka movement and resource selection.

### Conservation implications

Our study illustrates the complex anthropogenic and ecological processes that influence movement behavior of golden-crowned sifaka groups. We found evidence that human settlements and road networks play an important role in shaping sifaka foraging and ranging behavior. Additionally, ecological factors such as season are drivers of home range size and space use in this species. Our study enforces the importance of studying primate groups in both the rainy and dry seasons to ensure that conservation efforts meet the full range of a species’ movement, home range size, and resource needs. By understanding how forest type influences golden-crowned sifaka movement and foraging behavior, conservation management plans can be appropriately crafted to the unique forest types throughout the Loky-Manambato Protected Area (humid, moderate evergreen, dry deciduous, littoral, etc.), rather than the region as a whole. Our findings can also be used to inform Malagasy infrastructure and road development plans by working with local conservation NGOs, government officials, and construction teams to limit construction nearby lemur home ranges that are most impacted by human activity. We would advise that the national road not be re-routed towards Binara, the humid forest fragment, due to the strong avoidance lemurs display towards existing road networks and the increased movement of lemurs within this forest fragment. We detected the least avoidance of anthropogenic activity for lemurs in the moderate evergreen forest type, suggesting they are more resilient to the negative effects of human infrastructure. Overall, as anthropogenic disturbance continues to alter habitat structure throughout Madagascar, a deeper knowledge of how fragmentation, habitat loss, and infrastructure development influence golden-crowned sifaka space use, density, and population health will be essential for wildlife managers to make well informed decisions that improve conservation plans for at-risk species.

## Supplementary Information

**Additional file 1.** **Table S1.** Mean seasonal speed for golden-crowned sifaka (*Propithecus tattersalli*) groups. **Table S2.** Mean daily speed for golden-crowned sifaka (*Propithecus tattersalli*) groups. **Table S3.** GLM of golden-crowned sifaka (*Propithecus tattersalli*) foraging tree selection. **Table S4.** Formulation of the resource selection GLM of golden-crowned sifaka (*Propithecus tattersalli*) groups.

## Data Availability

The datasets used and/or analyzed during the current study are available from the corresponding author on reasonable request.
