# Supplementary material for: Environmental and anthropogenic influences on movement and foraging in a critically endangered lemur species, Propithecus tattersalli: implications for habitat conservation planning
Source: Mov Ecol. 2022 Apr 15;10:20. doi: 10.1186/s40462-022-00320-x (PMC9013159; doi:10.1186/s40462-022-00320-x)
Supplement: Supplementary file 1 — Additional file 1. Table S1. Mean seasonal speed for golden-crowned sifaka (Propithecus tattersalli) groups. Table S2. Mean daily speed for golden-crowned sifaka (Propithecus tattersalli) groups. Table S3. GLM of golden-crowned sifaka (Propithecus tattersalli) foraging tree selection. Table S4. Formulation of the resource selection GLM of golden-crowned sifaka (Propithecus tattersalli) groups. [file 40462_2022_320_MOESM1_ESM.docx]

Environmental and anthropogenic influences on movement and foraging in a critically endangered lemur species, *Propithecus tattersalli*: implications for habitat conservation planning

Meredith A. Semel (0000-0003-4317-8602), Heather N. Abernathy, Brandon P. Semel (0000-0003-3286-0382), Michael J. Cherry, Tsioriniaina J.C. Ratovoson, and Ignacio T. Moore

Supplemental Materials 1 – Supplemental Results

**Table S1.** Mean seasonal speed (meters/15 minutes) and corresponding confidence intervals for each golden-crowned sifaka (*Propithecus tattersalli*) group across each season monitored.

| Forest Fragment | Forest Type | Season | Forest Location | Mean Speed (meters/15-minutes) |
| --- | --- | --- | --- | --- |
| Bekaraoka | Moderate Evergreen | Dry | Interior | 60.2 (70.5, 49.9) |
| Bekaraoka | Moderate Evergreen | Rainy | Interior | 66.4 (78.1, 54.7) |
| Bekaraoka | Moderate Evergreen | Dry | Edge | 48.5 (57.1, 39.9) |
| Bekaraoka | Moderate Evergreen | Rainy | Edge | 68.8 (82.1, 55.5) |
| Binara | Humid | Dry | Interior | 42.6 (48.9, 36.3) |
| Binara | Humid | Rainy | Interior | 102.6 (119, 86.2) |
| Binara | Humid | Dry | Edge | 71.3 (84.3, 58.3) |
| Binara | Humid | Rainy | Edge | 94.6 (114.5, 74.7) |
| Solanamampilana | Dry Deciduous | Dry | Interior | 50.2 (58.1, 42.3) |
| Solanamampilana | Dry Deciduous | Rainy | Interior | 71 (81.5, 60.5) |
| Solanamampilana | Dry Deciduous | Dry | Edge | 67.4 (77.8, 57) |
| Solanamampilana | Dry Deciduous | Rainy | Edge | 97.3 (122.8, 71.8) |

**Table S2.** Mean daily speed (meters/15 minutes) and corresponding confidence intervals for each golden-crowned sifaka (*Propithecus tattersalli*) group across each day monitored.

| Forest Fragment | Forest Type | Season | Date | Mean daily speed (meters/15-minutes) (95% CI) |
| --- | --- | --- | --- | --- |
| Bekaraoka | Interior | dry | 6/7/2019 | 60.36 (41.86, 78.86) |
| Bekaraoka | Interior | dry | 6/8/2019 | 49 (33.8, 64.2) |
| Bekaraoka | Interior | dry | 6/9/2019 | 62.66 (42.96, 82.36) |
| Bekaraoka | Interior | dry | 6/10/2019 | 86.89 (32.59, 141.19) |
| Bekaraoka | Interior | dry | 6/11/2019 | 62.39 (40.29, 84.49) |
| Bekaraoka | Interior | dry | 6/12/2019 | 54.49 (35.09, 73.89) |
| Bekaraoka | Interior | dry | 6/13/2019 | 45.55 (32.95, 58.15) |
| Bekaraoka | Interior | rainy | 2/8/2019 | 64.87 (27.27, 102.47) |
| Bekaraoka | Interior | rainy | 2/9/2019 | 61.45 (30.95, 91.95) |
| Bekaraoka | Interior | rainy | 2/10/2019 | 70.89 (47.79, 93.99) |
| Bekaraoka | Interior | rainy | 2/11/2019 | 53.26 (31.26, 75.26) |
| Bekaraoka | Interior | rainy | 2/12/2019 | 79.95 (44.35, 115.55) |
| Bekaraoka | Interior | rainy | 2/13/2019 | 60.54 (31.64, 89.44) |
| Bekaraoka | Interior | rainy | 2/14/2019 | 76.41 (41.21, 111.61) |
| Bekaraoka | Interior | rainy | 2/15/2019 | 64.98 (27.68, 102.28) |
| Bekaraoka | Edge | dry | 6/16/2019 | 36.2 (23.4, 49) |
| Bekaraoka | Edge | dry | 6/17/2019 | 48.15 (32.55, 63.75) |
| Bekaraoka | Edge | dry | 6/18/2019 | 34.07 (23.87, 44.27) |
| Bekaraoka | Edge | dry | 6/19/2019 | 87.23 (39.93, 134.53) |
| Bekaraoka | Edge | dry | 6/20/2019 | 34.11 (16.51, 51.71) |
| Bekaraoka | Edge | dry | 6/21/2019 | 49.44 (29.94, 68.94) |
| Bekaraoka | Edge | dry | 6/22/2019 | 51.56 (35.76, 67.36) |
| Bekaraoka | Edge | rainy | 2/18/2019 | 27.06 (10.76, 43.36) |
| Bekaraoka | Edge | rainy | 2/19/2019 | 84.77 (59.67, 109.87) |
| Bekaraoka | Edge | rainy | 2/20/2019 | 74.88 (42.48, 107.28) |
| Bekaraoka | Edge | rainy | 2/21/2019 | 96.57 (58.97, 134.17) |
| Bekaraoka | Edge | rainy | 2/22/2019 | 82.34 (39.94, 124.74) |
| Bekaraoka | Edge | rainy | 2/23/2019 | 38.53 (18.73, 58.33) |
| Bekaraoka | Edge | rainy | 2/24/2019 | 39.74 (4.64, 74.84) |
| Binara | Interior | dry | 7/7/2019 | 43.38 (26.98, 59.78) |
| Binara | Interior | dry | 7/8/2019 | 47.27 (30.47, 64.07) |
| Binara | Interior | dry | 7/9/2019 | 39.67 (23.67, 55.67) |
| Binara | Interior | dry | 7/10/2019 | 37.75 (20.75, 54.75) |
| Binara | Interior | dry | 7/11/2019 | 36.01 (20.51, 51.51) |
| Binara | Interior | dry | 7/12/2019 | 43.39 (27.39, 59.39) |
| Binara | Interior | dry | 7/13/2019 | 51.39 (34.19, 68.59) |
| Binara | Interior | rainy | 3/2/2019 | 115.4 (55.1, 175.7) |
| Binara | Interior | rainy | 3/3/2019 | 79.34 (50.64, 108.04) |
| Binara | Interior | rainy | 3/4/2019 | 121.08 (69.98, 172.18) |
| Binara | Interior | rainy | 3/5/2019 | 125.12 (78.82, 171.42) |
| Binara | Interior | rainy | 3/6/2019 | 88.47 (57.17, 119.77) |
| Binara | Interior | rainy | 3/8/2019 | 111.58 (65.28, 157.88) |
| Binara | Interior | rainy | 3/9/2019 | 91.19 (46.09, 136.29) |
| Binara | Interior | rainy | 3/10/2019 | 90.42 (50.32, 130.52) |
| Binara | Edge | dry | 7/15/2019 | 80.07 (49.27, 110.87) |
| Binara | Edge | dry | 7/16/2019 | 69.74 (30.04, 109.44) |
| Binara | Edge | dry | 7/17/2019 | 60.45 (22.05, 98.85) |
| Binara | Edge | dry | 7/18/2019 | 82.4 (49.8, 115) |
| Binara | Edge | dry | 7/19/2019 | 75.27 (45.07, 105.47) |
| Binara | Edge | dry | 7/20/2019 | 69.02 (30.82, 107.22) |
| Binara | Edge | dry | 7/21/2019 | 64.88 (35.28, 94.48) |
| Binara | Edge | rainy | 3/12/2019 | 116.35 (27.65, 205.05) |
| Binara | Edge | rainy | 3/13/2019 | 73.22 (39.92, 106.52) |
| Binara | Edge | rainy | 3/14/2019 | 75.98 (48.08, 103.88) |
| Binara | Edge | rainy | 3/15/2019 | 139.56 (79.96, 199.16) |
| Binara | Edge | rainy | 3/16/2019 | 72.59 (45.89, 99.29) |
| Binara | Edge | rainy | 3/17/2019 | 73.27 (48.37, 98.17) |
| Binara | Edge | rainy | 3/18/2019 | 124.79 (44.49, 205.09) |
| Solanamampilana | Interior | dry | 7/28/2019 | 67.4 (45.8, 89) |
| Solanamampilana | Interior | dry | 7/29/2019 | 48.48 (35.28, 61.68) |
| Solanamampilana | Interior | dry | 7/30/2019 | 77.09 (57.39, 96.79) |
| Solanamampilana | Interior | dry | 7/31/2019 | 56.02 (38.92, 73.12) |
| Solanamampilana | Interior | dry | 8/1/2019 | 57.12 (37.52, 76.72) |
| Solanamampilana | Interior | dry | 8/2/2019 | 39.52 (10.62, 68.42) |
| Solanamampilana | Interior | dry | 8/3/2019 | 16.12 (1.98, 34.22) |
| Solanamampilana | Interior | rainy | 3/29/2019 | 58.65 (16.15, 101.15) |
| Solanamampilana | Interior | rainy | 3/30/2019 | 68.66 (47.76, 89.56) |
| Solanamampilana | Interior | rainy | 3/31/2019 | 91.8 (66.1, 117.5) |
| Solanamampilana | Interior | rainy | 4/1/2019 | 69.57 (46.57, 92.57) |
| Solanamampilana | Interior | rainy | 4/2/2019 | 63.81 (44.91, 82.71) |
| Solanamampilana | Interior | rainy | 4/3/2019 | 63.39 (39.29, 87.49) |
| Solanamampilana | Edge | dry | 8/5/2019 | 53.9 (28.3, 79.5) |
| Solanamampilana | Edge | dry | 8/6/2019 | 69.1 (32.2, 106) |
| Solanamampilana | Edge | dry | 8/7/2019 | 70.64 (40.94, 100.34) |
| Solanamampilana | Edge | dry | 8/8/2019 | 67.24 (46.64, 87.84) |
| Solanamampilana | Edge | dry | 8/9/2019 | 76.92 (52.52, 101.32) |
| Solanamampilana | Edge | dry | 8/10/2019 | 77.43 (49.43, 105.43) |
| Solanamampilana | Edge | dry | 8/11/2019 | 52.9 (25.1, 80.7) |
| Solanamampilana | Edge | rainy | 4/6/2019 | 85.95 (56.25, 115.65) |
| Solanamampilana | Edge | rainy | 4/7/2019 | 105.04 (42.44, 167.64) |
| Solanamampilana | Edge | rainy | 4/8/2019 | 81.63 (58.33, 104.93) |
| Solanamampilana | Edge | rainy | 4/9/2019 | 195.01 (78.01, 312.01) |
| Solanamampilana | Edge | rainy | 4/10/2019 | 58.49 (40.19, 76.79) |
| Solanamampilana | Edge | rainy | 4/11/2019 | 54.71 (38.11, 71.31) |

**Table S3.** GLM of golden-crowned sifaka (*Propithecus tattersalli*) foraging tree selection based on occupied forest type. Columns indicate the number of parameters (K), the relative difference in AICc values compared to the top ranked model (ΔAICc), the AICc weights (W), and the log-likelihood (LL) of the model-selection procedure examining foraging tree selection of lemurs based on occupied forest type (dry, moderate, and humid). Model dispersion was examined by calculating the sum of squared Pearson (SSQ) residuals, the ratio of (SSQ residuals/ residual degrees-of-freedom), the residual df, and the p-value based on the appropriate χ2 distribution. Goodness-of-model fit was examined by calculating a conditional and marginal R^2^. The marginal R^2^ considered only the variance of the fixed effects, while the conditional R^2^ took both the fixed and random effects into account. Model variables included- CV: Crown Volume, TBA: Tree basal area, V: Distance to village, R: Distance to roads, F: Distance to forest edge, FT: Forest type.

| Model Formulation | K | AICc | ΔAICc | W | LL | Sum of squared Pearson (SSQ) residuals | Ratio of (SSQ residuals/residual degrees-of-freedom) | Residual df | P-value based on the appropriate χ^2^ distribution | Marginal R^2^ | Conditional R^2^ |
| --- | --- | --- | --- | --- | --- | --- | --- | --- | --- | --- | --- |
| Dry Forest | | | | | | | | | | | |
| (CV+ TBA + V + R + F)*Season | 13 | 4085.42 | 0.00 | 1.00 | -2029.71 | 5597.49 | 0.89 | 6311 | 1.00 | 0.275 | 0.535 |
| CV+ TBA + V + R + F | 7 | 4105.11 | 19.7 | 0.00 | -2045.56 | 5711.05 | 0.90 | 6317 | 1.00 | 0.252 | 0.584 |
| Moderate Forest | | | | | | | | | | | |
| (CV+ TBA + V + R + F)*Season | 13 | 2944.24 | 0.0 | 1.00 | -1459.12 | 2747.54 | 0.88 | 3120 | 0.9999995 | 0.453 | 0.630 |
| CV+ TBA + V + R + F | 7 | 3043.27 | 99.04 | 0.00 | -1514.64 | 2848.85 | 0.91 | 3126 | 0.9998445 | 0.349 | 0.424 |
| Humid Forest | | | | | | | | | | | |
| (CV+ TBA + V + R + F)*Season | 13 | 4158.31 | 0.00 | 1.00 | -2066.15 | 5149.59 | 0.86 | 5975 | 1.00 | 0.402 | 0.644 |
| CV+ TBA + V + R + F | 7 | 4206.98 | 48.68 | 0.00 | -2096.49 | 5110.32 | 0.85 | 5981 | 1.00 | 0.379 | 0.537 |

**Table S4**. Formulation of the resource selection GLM models of golden-crowned sifaka (*Propithecus tattersalli*) broken up by forest type and season. Overdispersion was tested using a chi-squared comparing the model deviance by the residual degrees-of-freedom. Values greater than one for a dispersion statistic indicate overdispersion. Goodness-of-model fit was examined by calculating a pseudo R^2^ for each model. Model variables included- CV: Crown Volume, TBA: Tree basal area, V: Distance to village, R: Distance to roads, F: Distance to forest edge.

| Data | Model | Pearson Chi^2^ | dispersion statistic | pseudo R^2^ |
| --- | --- | --- | --- | --- |
| Humid Forest, Wet Season | CV + TBA + V + R + F | 3626.23 | 0.86 | 0.33 |
| Humid Forest, Dry Season | CV + TBA + V + R + F | 1958.66 | 0.93 | 0.25 |
| Moderate Forest, Wet Season | CV + TBA + V + R + F | 1674.30 | 0.84 | 0.38 |
| Moderate Forest, Dry Season | CV + TBA + V + R + F | 1124.53 | 1.00 | 0.10 |
| Dry Forest, Wet Season | CV + TBA + V + R + F | 3305.44 | 0.82 | 0.26 |
| Dry Forest, Dry Season | CV + TBA + V + R + F | 1937.99 | 0.98 | 0.12 |
